# Supplementary material for: Novel lytic phages improve the antibiofilm activity of dalbavancin, daptomycin, and fosfomycin against vancomycin-resistant enterococci
Source: Microbiol Spectr. 2025 Nov 19;14(1):e01818-25. doi: 10.1128/spectrum.01818-25 (PMC12772233; doi:10.1128/spectrum.01818-25)
Supplement: Supplemental material — Table S1; Fig. S1 and S2. [file spectrum.01818-25-s0001.pdf]

Table S1. Microcalorimetry analysis of *E. faecium* and *E. faecalis* biofilm treated with phages alone, antibiotics alone at different concentrations (1×, 10× or 100× MIC) and in phage-antibiotic combinations. Data is expressed as mean ± SD.

		E. faecium								***E. faecalis***							
		HF_{Max} (µW)				t_{Max} (h)				HF_{Max} (µW)				t_{Max} (h)			
		ABX	*p* vs. GC	ABX + CUB-FM	*p* vs. GC	ABX	*p* vs. GC	ABX + CUB-FM	*p* vs. GC	ABX	*p* vs. GC	ABX + CUB-FS	*p* vs. GC	ABX	*p* vs. GC	ABX + CUB-FS	*p* vs. GC
**Fosfomycin**	x1MIC	152.3 ± 3.7	0.004	117.7 ± 6.7	<0.001	6.5 ± 1.2	0.431	12.6 ± 1.6	<0.001	187.8 ± 2.5	>0.999	76.9 ± 6.7	<0.001	4.2 ± 1.0	0.227	13.8 ± 1.0	0.003
	x10MIC	160.8 ± 5.6	0.060	122.3 ± 4.9	<0.001	9.9 ± 1.5	<0.001	14.2 ± 1.5	<0.001	184.3 ± 2.5	>0.999	-	-	5.4 ± 0.1	0.054	-	-
	x100MIC	151.0 ± 6.8	0.002	117.7 ± 6.3	<0.001	11.9 ± 1.7	<0.001	14.6 ± 1.5	<0.001	163.7 ± 9.3	0.762	-	-	6.0 ± 0.0	0.007	-	-
**Daptomycin**	x1MIC	204.5 ± 3.2	0.755	127.7 ± 5.8	<0.001	5.7 ± 1.2	0.870	12.1 ± 1.0	<0.001	184.3 ± 1.6	>0.999	103.6 ± 5.8	<0.001	3.9 ± 0.5	0.431	7.6 ± 0.9	<0.001
	x10MIC	173.2 ± 2.5	0.001	120.7 ± 6.3	<0.001	8.2 ± 1.9	<0.001	15.0 ± 1.4	<0.001	168.6 ± 4.5	0.871	71.3 ± 5.5	<0.001	4.9 ± 0.2	0.059	11.0 ± 1.3	<0.001
	x100MIC	147.6 ± 1.9	<0.001	117.3 ± 5.8	<0.001	12.5 ± 1.2	<0.001	20.6 ± 1.4	<0.001	146.5 ± 4.1	<0.070	70.1 ± 5.4	<0.001	10.6 ± 1.2	0.002	25.7 ± 1.1	<0.001
**Dalbavan**	x1MIC	173.8 ± 2.2	0.422	114.60 ± 4.9	<0.001	8.8 ± 1.3	<0.001	10.4 ± 1.5	<0.001	181.4 ± 1.8	>0.999	91.0 ± 5.9	<0.001	3.7 ± 0.0	0.876	7.7 ± 1.3	<0.001
	x10MIC	146.6 ± 3.1	0.01	109.0 ± 7.1	<0.001	11.8 ± 1.5	<0.001	13.5 ± 1.7	<0.001	171.8 ± 2.9	0.851	-	-	3.9 ± 0.0	0.431	-	-

x100MIC	86.8 ± 1.7	<0.001	45.0 ± 7.6	<0.001	19.0 ± 1.8	<0.001	22.3 ± 1.3	<0.001	62.3 ± 3.0	<0.001	-	-	15.0 ± 1.5	<0.001	-	-

	**GC**	**CUB-FM**	*p*	**GC**	**CUB-FS**	*p*
HF_{Max} (μW)	210.4 ± 6.5	121.5 ± 1.6	<0.001	181.6 ± 5.3	106.3 ± 4.3	0.002
t_{Max} (h)	5.2 ± 1.0	11.5 ± 1.3	<0.001	3.6 ± 1.1	6.6 ± 1.1	0.004

HF_{Max}, maximum heat flow peak; t_{Max}, time to reach maximum heat; GC, growth control; ABX, antibiotics.

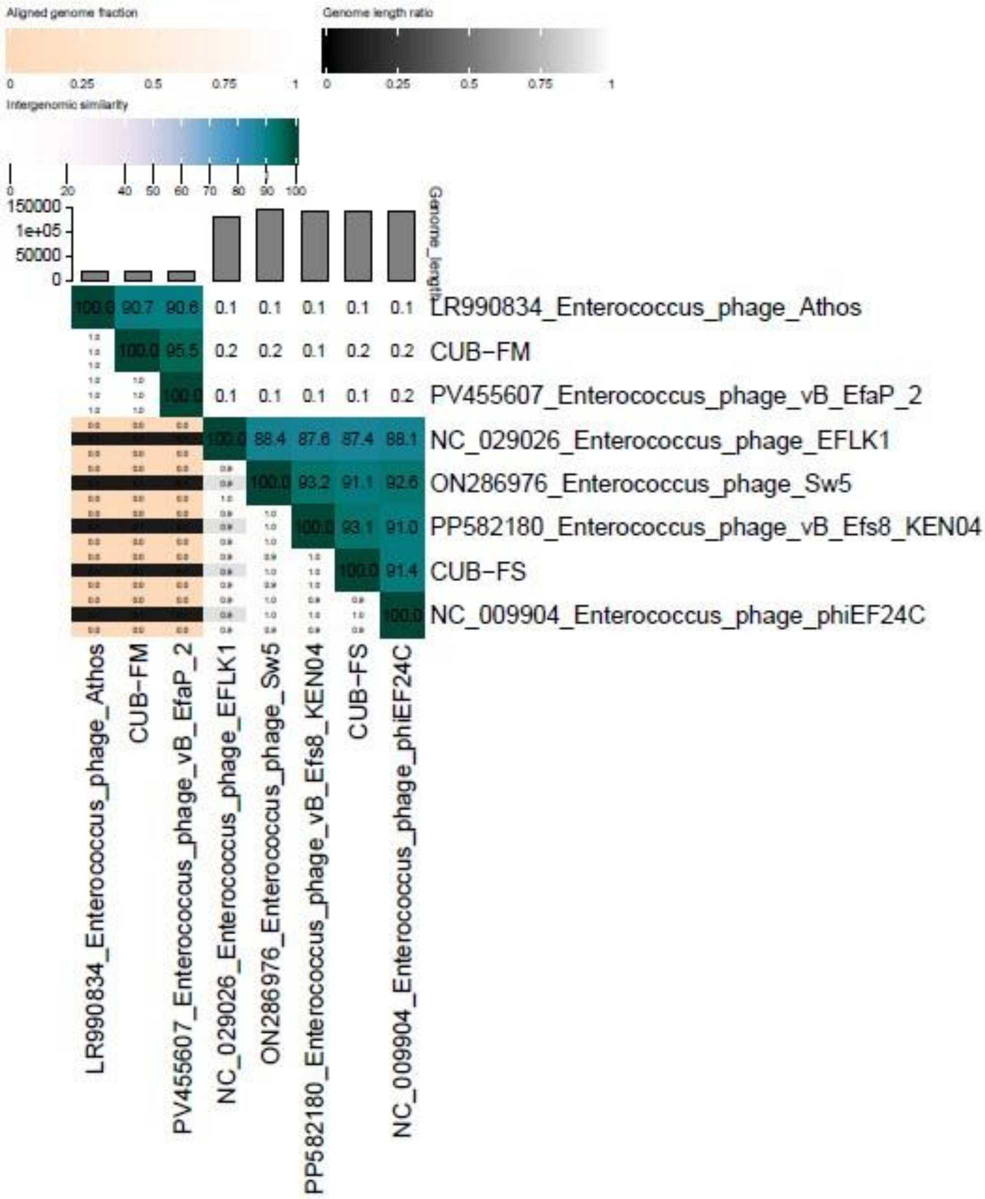

Fig. S1 Intergenomic similarity heatmap of enterococcal phages generated with VIRIDIC.

The heatmap depicts pairwise intergenomic similarities among the newly isolated phages CUB-FM and CUB-FS and representative reference enterococcal phages. Colors correspond to the percentage of genome-wide nucleotide identity as calculated by VIRIDIC. CUB-FM clustered closely with *Enterococcus* phage Athos (92.6% identity), supporting its classification within the *Salasmaviridae* family. CUB-FS showed 98.6% identity with *Enterococcus* phage phiEF24C, consistent with assignment to the *Herelleviridae* family. Genome length, aligned genome fraction, and genome length ratios are shown below the heatmap for comparison.

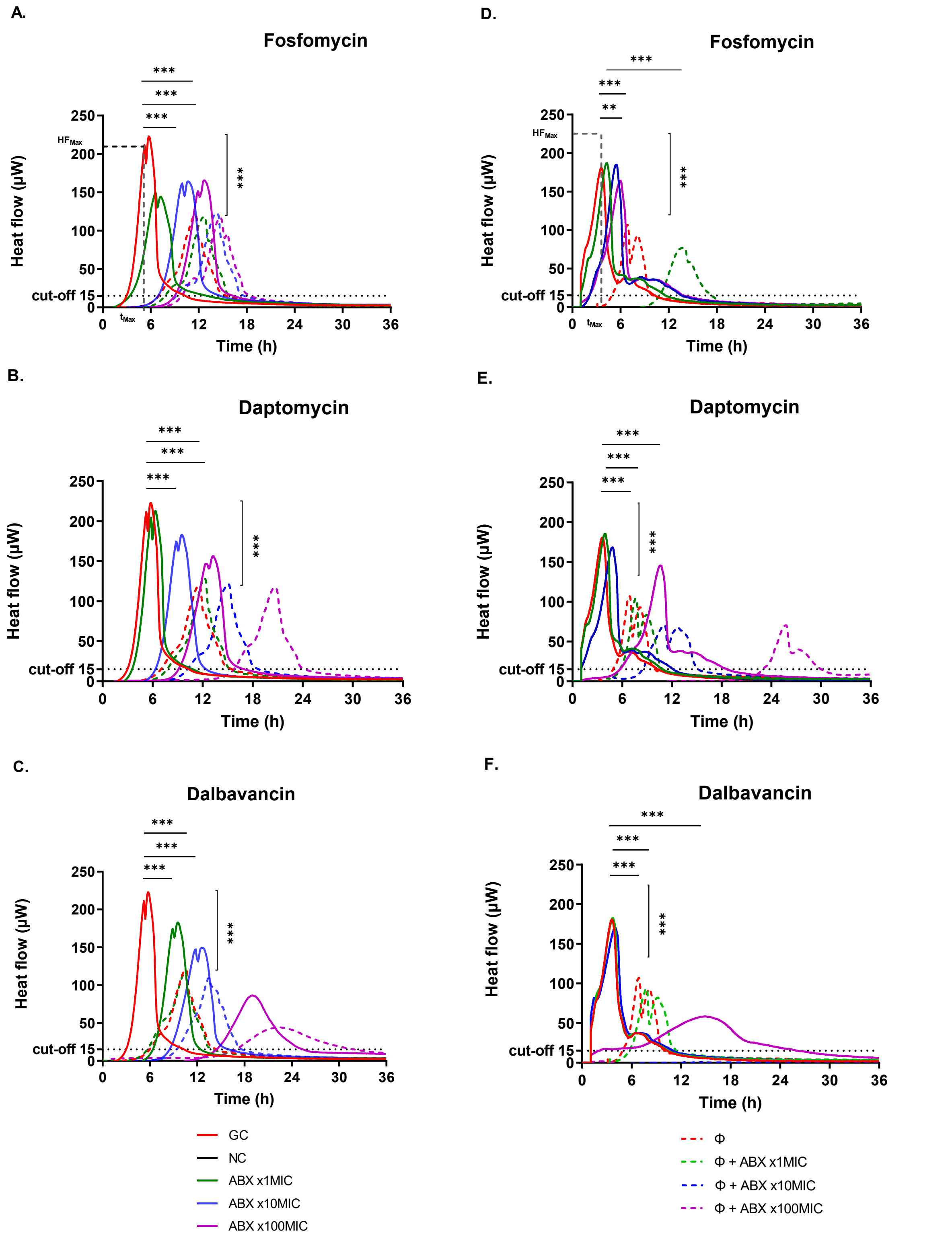

Fig. S2. Microcalorimetry analysis of *E. faecium* (A – C) and *E. faecalis* (E – F) biofilm treated with phages alone and in combination with antibiotics at different concentrations (1×, 10× or

100× MIC). GC, growth control; NC, negative control; ABX, antibiotics; Φ, phage (10^{12} PFU/ml CUB-FM for *E. faecium* and 10^{8} PFU/ml CUB-FS for *E. faecalis*). Dashed lines represent time to 15 μW heat flow, i.e. thermal growth onset time. This figure is included to illustrate the raw data profile and baseline curve shapes used for downstream Δt_{Max} and ΔHF_{Max} calculations. Data shown are from one representative replicate.
